# Advancements in the Treatment of Triple-Negative Breast Cancer: A Narrative Review of the Literature

**DOI:** 10.7759/cureus.21970

**Published:** 2022-02-07

**Authors:** Ian Landry, Vikram Sumbly, Mallorie Vest

**Affiliations:** 1 Medicine, Icahn School of Medicine at Mount Sinai, New York City Health and Hospitals/Queens, Jamaica, USA; 2 Internal Medicine, Icahn School of Medicine at Mount Sinai, New York City Health and Hospitals/Queens, Jamaica, USA

**Keywords:** radiation, therapeutics, breast cancer, chemotherapy, triple negative breast cancer

## Abstract

Triple-negative breast cancers (TNBCs) are aggressive tumors that are more common in young women, African American populations, and those with hereditary mutations. These tumors are notable for their high recurrence rate and predilection for chemoresistance. The goal of this narrative review is to describe the current treatment options for patients diagnosed with TNBC and to review the studies that have put forward these recommendations. We searched PubMed and Cochrane databases for free full-text, English-language studies published within the last several years pertaining to the search items “triple negative breast cancer” and “treatment”. We included clinical trials and retrospective reviews that had clear designs and assessed their findings against a gold standard or placebo and included evidence of overall response and/or survival outcomes.

Patients with early-stage (I-III) TNBC still benefit from treatment with chemotherapeutic regimens involving anthracyclines, taxanes, and antimetabolites. Platinum-based therapies have been shown to improve the overall pathologic complete response (pCR), but there is conflicting evidence with regard to their contribution to disease-free survival (DFS) and overall survival (OS), even with the addition of a poly (ADP-ribose) polymerase (PARP) inhibitor. Patients with residual disease after neoadjuvant chemotherapy and surgical intervention have shown a significant improvement in OS when treated with adjuvant capecitabine. The high mutation burden in metastatic TNBC (mTNBC) allows for targeted therapies and immune checkpoint inhibitors. mTNBCs that express programmed death ligand-1 (PD-L1) receptors may achieve improved response and survival if their regimen includes a monoclonal antibody. Antibody-drug conjugates (ADCs) can deliver high doses of chemotherapy and significantly impact survival in mTNBC regardless of the level of biomarkers expressed by the tumor cells. PARP inhibitors significantly improve survival in newly diagnosed, treatment-naive mTNBC, but have shown mixed results in patients with a history of previous therapy. PARP inhibitors may also target patients with somatic breast cancer (BRCA) and partner and localizer of BRCA-2 (PALB2) mutations, which would allow for more options in this subset of patients. While other rare targets have shown mixed results, the future of treatment may lie in anti-androgen therapy or the development of cancer vaccinations that may increase the immunogenicity of the tumor environment.

The management of TNBC includes treatment with multimodal chemotherapy, immune checkpoint inhibitors, and ADCs. The optimal approach depends on a multitude of factors, which include the stage of the tumor, its unique mutational burden, comorbid conditions, and the functional status of the patient. Physicians should be familiar with the advantages and disadvantages of each therapy in order to appropriately counsel and guide their patients.

## Introduction and background

Advancements in genomic techniques have improved the assessment and diagnosis of breast cancer, enabling the classification of the condition into four molecular subtypes based on the predominant genes [1,2]. Triple-negative breast cancers (TNBCs) are a heterogenous subgroup characterized by their lack of expression of estrogen receptor (ER), progesterone receptor (PR), and human epidermal growth factor receptor 2 (HER2). TNBC is unique in that its lack of receptor expression often portends a poorer prognosis and poses unique therapeutic challenges. The variation in its gene expression has created a dynamic tumor microenvironment, which is often associated with chemoresistance, aggressive behavior, and frequent recurrence [3,4].

Breast cancer remains the most common cancer diagnosed in women, with an overall lifetime risk of 13% [5]. In 2021, 282,000 new cases of breast cancer were diagnosed, with approximately 13.1 new cases of TNBC per 100,000 women [5]. TNBC carries a higher mortality risk with a relative five-year survival of 65% in regional disease and a dismal 12% when diagnosed at a distant stage [5]. Its aggressive nature often leads to early recurrence rates within the first three to five years with a predilection for the central nervous system and lungs [3]. Overwhelmingly, black women experience a higher incidence of TNBC and a disproportionately higher mortality rate [6]. Women aged less than 35 years make up a significant proportion of TNBC patients, and in them, the condition may present as a poorly differentiated, aggressive tumor with multiple germline mutations [7,8,9].

There is no clear consensus on the standardization of care for TNBC, and research on this group of patients has been limited due to poor outcomes. The goal of our review is to compile and condense the most recent and relevant studies to present a clear picture of the current options for the treatment of this underserved population.

## Review

Methods

We searched PubMed and Cochrane databases with the addition of ClinicalTrials.gov for ongoing and future research. The keywords included “triple negative breast cancer”, “metastatic triple negative breast cancer”, “treatment”, and “guidelines”. The inclusion criteria for evaluation included publications that involved only basic research, clinical research, and translational research papers written in English. Case reports and case series were excluded. Since the goal was to obtain a consensus on the effects of treatment as it relates to patient outcomes, clinical trials that evaluated new therapies against standard chemotherapy or placebo were specifically sought out. Studies that had information on pathologic complete response (pCR), disease-free survival (DFS), and/or overall survival (OS) were included. Exclusion criteria were as follows: those studies that did not contain an appropriate control group, such as ones involving placebo or the current standard of care. In total, 1,246 records were assessed and analyzed. Studies that met the inclusion criteria were compiled into a table and classified based on their assessment of chemotherapy and immunotherapy. The co-authors assessed eligible studies in the same manner and any disputes between reviewers were deliberated to mutual satisfaction. Outcomes of interest included overall response rate (ORR), DFS or progression-free survival (PFS), and OS. These outcomes were extracted and are displayed in Tables 1, 2.

**Table 1:** Current chemotherapy options for triple-negative breast cancer pCR: pathologic complete response; TNBC: triple-negative breast cancer; DFS: disease-free survival; OS: overall survival; CTx: chemotherapy; PM: paclitaxel/non-pegylated liposomal doxorubicin; Bev: bevacizumab (Avastin); Cbp: carboplatin; TAC: docetaxel (Taxotere), doxorubicin (Adriamycin), cyclophosphamide; HR: hazard ratio

Study	Population	Regimen	Complete response (CR)	P-value	Disease-free survival (DFS)	P-value	Overall survival (OS)	P-value
Chemotherapy trials								
Green et al. (2005) [10]	I-IIIA TNBC without previous treatment (n=258)	Weekly paclitaxel	48%	0.007	NA	NA	NA	NA
		Every 3 weeks	23%		NA		NA	
GeparSixto, von Minckwitz et al. (2014) [11]	II-III TNBC without previous treatment (n=315)	PM + Bev	36.9%	0.005	76%	0.035, HR: 0.56 (0.33-0.96)	NA	NA
		PM + Bev + Cbp	53.2%		86%		NA	
CALGB 40603 (Alliance), Sikov et al. (2014) [12]	II-III TNBC without previous treatment (n=433)	T-AC + Bev	46%	0.0018	71%	0.36, HR: 0.84 (0.58-1.22)	NA	NA
		T-Cbp-AC + Bev	60%		76%		NA	
BrighTNess, Loibl et al. (2018) [13]	II-III TNBC without previous treatment (n=634)	T-AC	31%	<0.001	68.5%	HR: 0.58 (0.39-0.87)	13.9%	NA
		T-Cbp-AC	58%		79.3%		10%	
		T/Cbp-V-AC	53%		78.2%		12%	
CREATE-X, Masuda et al. (2017) [14]	I-III TNBC who did not achieve a pCR after NAC/surgery (n=286, 32% of total population)	Control	NA	NA	56.1%	HR: 0.58 (0.39-0.87)	70.3%	HR: 0.52 (0.30-0.90)
		Capecitabine	NA		69.8%		78.8%	
GEICAM, Lluch et al. (2019) [15]	II-III TNBC previously treated with anthracycline +/- taxane (n=448)	Observation	NA	NA	76.8%	HR: 0.82 (0.63-1.06)	85.9%	HR: 0.92 (0.66-1.28)
		Capecitabine	NA		79.6%		86.2%	
Zhang et al. (2015) [16]	mTNBC previously treated with at least 1 line of CTx (n=379)	Platinum CTx	NA	NA	7.8 months	<0.001	19.6 months	0.82
		Non-platinum CTx	NA		4.9 months		19.2 months	

**Table 2:** Current immunotherapy options for triple-negative breast cancer *Chemotherapy options included nab-paclitaxel, paclitaxel, or gemcitabine/carboplatin. **TPC (treatment of physician’s choice) included capecitabine, vinorelbine, or eribulin pCR: pathologic complete response; TNBC: triple-negative breast cancer; DFS: disease-free survival; OS: overall survival; CTx: chemotherapy; Cbp: carboplatin; Nab-Pac: nab-paclitaxel; pembro: pembrolizumab; EC: epirubicin/cyclophosphamide; GC: gemcitabine/carboplatin

Study	Population	Regimen	Complete response (CR)	P-value	Disease-free survival (DFS)	P-value	Overall survival (OS)	P-value
Immunotherapy trials								
GeparNuevo, Loibl et al. (2019) [17]	II-III TNBC without previous treatment (n=174)	CTx only	41.40%	0.035, OR: 2.22 (1.06-4.64)	79.50%	0.015, HR: 0.37 (0.15-0.87)	NA	NA
		Duravalumab + Nab-Pac + EC	61%		91.40%		NA	
NEOTRIPAPDL1, Bianchini (2020) [18]	Early high-risk or locally advanced TNBC (n=280)	Placebo + Cbp/Nab-Pac	55%	0.148	NA	NA	NA	NA
		Atezolizumab + Cbp/Nab-Pac	32%					
Keynote-355, Schmid et al. (2020) [19]	Previously untreated mTNBC (PD-L1+) (n=847)	Pembro + CTx*	NA	NA	9.7 months	<0.001, HR: 0.65	NA	NA
		Placebo + CTx	NA		5.6 months		NA	
					NA		NA	
IMpassion130, Emens et al. (2021) [20]	mTNBC or unresectable TNBC without prior treatment or completed neoadjuvant CTx longer than 12 months (PD-L1+) (n=396)	Placebo + Nab-Pac	NA	NA	5.3 months	<0.05, HR: 0.63 (0.50-0.80)	18 months	<0.05, HR: 0.71 (0.54-0.93)
		Atezolizumab + Nab-Pac	NA		7.5 months		25 months	
IMpassion131, Miles et al. (2021) [21]	mTNBC without prior treatment or completed neoadjuvant CTx longer than 12 months (PD-L1+) (n=333)	Placebo + Pac	NA	NA	22.1 months	HR: 1.12 (0.76-1.65)	NA	NA
		Atezolizumab + Pac + DEX	NA		28 months		NA	
O’Shaughnessy et al. (2011) [22]	mTNBC or locally recurrent who received no more than 2 previous CTx regimens (n=116)	GC alone	32%	<0.02	3.6 months	HR: 0.59 (0.39-0.90)	7.7 months	HR: 0.57 (0.36-0.90)
		GC + Iniparib	52%		5.9 months		12.3 months	
EMBRACA, Litton et al. (2019) [23]	BRCA+/HER2- mTNBC who received no more than 3 lines of previous CTx (n=412)	Talazoparib	NA	NA	8.6 months	<0.001, HR: 0.54 (0.41-0.71)	19.3 months	0.17, HR: 0.85 (0.67-1.07)
		TPC**	NA		5.6 months		19.5 months	
OlympiAD, Robson et al. (2019) [24]	BRCA+/HER2- mTNBC who received no more than 2 lines of previous CTx (n=205)	Olaparib	NA	NA	NA	NA	22.6 months	0.02, HR: 0.51 (0.29-0.90)
		TPC**	NA		NA		14.7 months	

Key findings

Seven of the included studies [10-16] evaluated chemotherapeutic options in TNBC, while eight studies [17-24] evaluated advances in immunotherapy.

Early-Stage TNBC results

**Figure 1:**
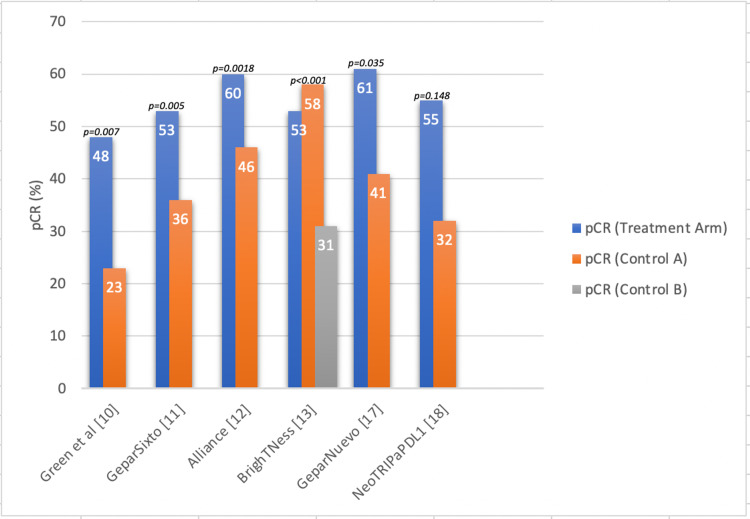
Pathologic complete response (pCR) in early-stage triple-negative breast cancer* *[10-13,17,18]

Green et al. [10] compared weekly vs. every-three-week dosing of paclitaxel, and five of the studies contained treatment arms that evaluated carboplatin use with or without immunotherapy or targeted treatment. Five of the six studies (Figure 1) found a greater complete response in the treatment group when compared to the controls (placebo vs. current guideline of care). One study did not find a statistically significant difference between the treatment groups while the findings of other studies were all statistically significant.

**Figure 2:**
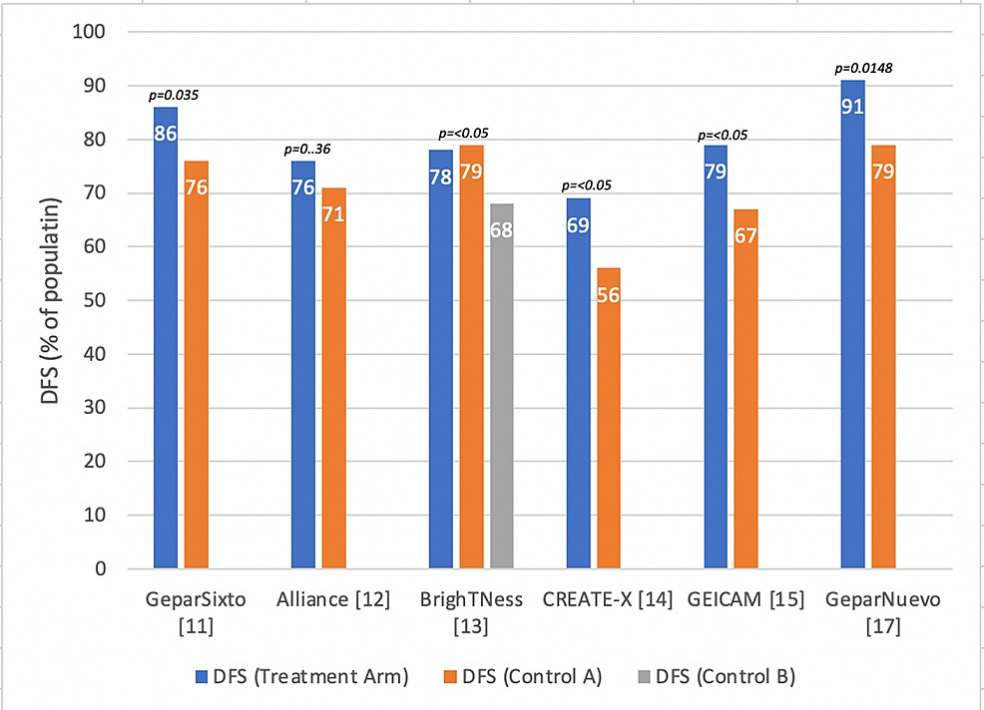
Disease-free survival (DFS) in early-stage triple-negative breast cancer* *[11-15,17]

Four of these original studies also had information on DFS. Two additional studies that had information on survival were included and are visualized in Figure 2. Five of the studies found significant differences between the treatment group and placebo or controls. Four of these had significantly improved DFS.

Metastatic TNBC (mTNBC)

**Figure 3:**
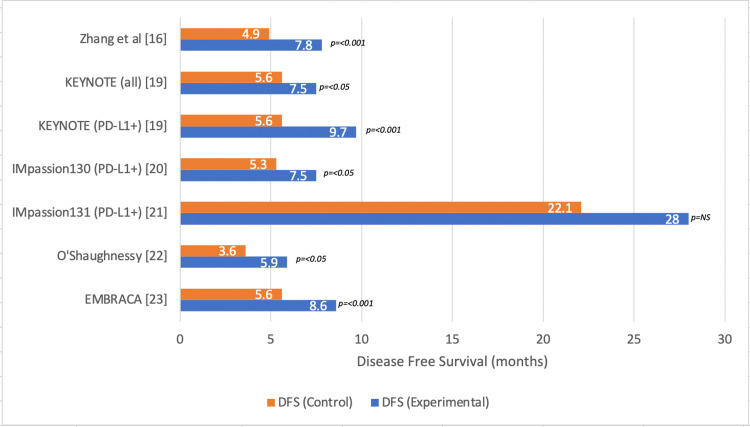
Disease-free survival (DFS) in metastatic triple-negative breast cancer* *[16,19-23]

**Figure 4:**
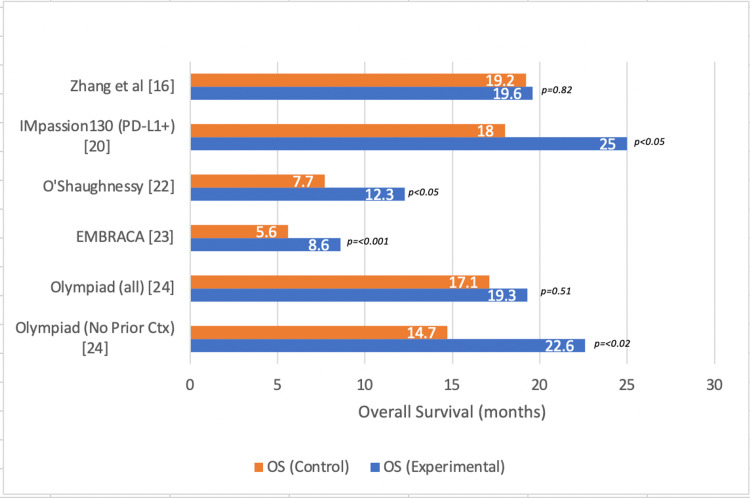
Overall survival (OS) in metastatic triple-negative breast cancer* *[16,20,22-24]

Survival outcomes were evaluated in six different studies. One of the studies compared platinum versus non-platinum chemotherapy, two of the studies evaluated the use of poly (ADP-ribose) polymerase (PARP) inhibitors, and three of the studies evaluated the effects of immunotherapy in mTNBC (Figure 3). All studies showed improved DFS in the treatment arm compared to controls or placebo with only one study finding insignificant results [IMPASSION131]. OS was significantly improved in the treatment arms of four separate studies included in the analysis (Figure 4).

Discussion

Current Treatment Options 

The current treatment protocol includes the common chemotherapeutic groups: anthracyclines, alkylating agents, anti-microtubule agents, and antimetabolite agents [5]. Early-stage (I-III) TNBC patients are often candidates for neoadjuvant chemotherapy. In contrast, metastatic TNBC (mTNBC) is an aggressive subtype with poorer disease-specific survival than hormone receptor-positive subtypes, with a median survival of one year [5]. The prevalence of TNBC is higher in young African American females with an average age at diagnosis of 53 years [5]. TNBC has a strong correlation with BRCA mutation with nearly 20% of patients testing positive for the germline mutation [5]. The advancement of targeted therapies, immune checkpoint inhibitors, and antibody-drug conjugates (ADCs) holds promise in the improvement of PFS and OS.

Taxanes and Anthracyclines

Taxanes and anthracycline-based chemotherapeutic regimens are the mainstays of TNBC treatment [5]. In the neoadjuvant setting, the addition of taxanes (T) to Adriamycin-cyclophosphamide (AC) regimens incrementally improves the proportion of patients who achieved pCR [5]. Green et al. [10] found that weekly paclitaxel use in TNBC patients led to a marked improvement in pCR compared to those who were treated every three weeks (48% vs. 23%, p=0.007).

Platinum-Based Therapy

The GeparSixto trial [17] was a phase II trial that evaluated the neoadjuvant use of standard chemotherapy with the addition of a carboplatin regimen. Patients were treated with paclitaxel and non-pegylated liposomal doxorubicin with or without carboplatin. The pCR was significantly improved in the carboplatin group (53% vs. 36%, p=0.005). However, the use of this regimen was associated with higher toxicity, with less than 60% of the population able to tolerate the complete course of treatment. At three-year follow-up, DFS was also improved in the carboplatin group (86%) vs. the non-carboplatin group (76%) with a hazard ratio of 0.56 (0.33-0.96), p=0.035.

The CALGB40603/Alliance trial [12] randomized patients with stage II or III TNBC to four treatment arms [T-AC, T-AC (Bev), T/carbo-AC, T/carbo-AC (Bev)]. When stratifying the patients by carboplatin (n=221) vs. no carboplatin (n=212), the pCR was significantly better in the carboplatin group (60% vs. 46%, p=0.0018). In this trial, toxicity also affected the completion of the therapy with fewer patients able to complete their prescribed regimen. Although the pCR was statistically different, it was not interpreted as clinically significant as the overall event-free survival (EFS) was similar [carboplatin: 76%, non-carboplatin: 71%; HR: 0.84 (0.58-1.22), p=0.36].

While it was clear that platinum could increase the pCR, its addition remained questionable based on the conflicting survival results from these trials. The BrighTNess trial [13], which randomized stage II-III TNBC patients into three treatment arms, evaluated whether the addition of a PARP inhibitor (veliparib) to platinum-based therapy would further increase the pCR. The pCR was not statistically different between the groups. The four-year EFS for the combined paclitaxel-carboplatin-veliparib group was 78.2% vs. 79.3% in the paclitaxel-carboplatin group, and 68.5% in the paclitaxel-only group. The triple combination was associated with a significantly higher EFS than paclitaxel alone (HR: 0.63, p=0.016); however, it was not superior to the combined paclitaxel-carboplatin group (HR: 1.12, p=0.62). Overall, the mortality rate was low in all three groups: 10% (carboplatin group), 12% (triple combination group), and 13.9% in the paclitaxel-only group.

Antimetabolites

CREATE-X [14] studied stage I-III breast cancer patients who did not achieve a pCR with neoadjuvant therapy (NAC) and surgery. These patients were then randomized to a control group or adjuvant-therapy group with capecitabine. Among patients with TNBC who received capecitabine (n=286), the DFS was significantly improved [HR: 0.58 (0.39-0.87)] as well as OS [HR: 0.52 (0.30-0.90)]. This led to the justification of its use in TNBC patients with residual disease. Conversely, the GEICAM trial [15] randomized stage II-III patients who had previously been treated with anthracyclines with or without taxanes into capecitabine vs. observation-only. The DFS was not significantly higher in the capecitabine group (79.6%) vs. the observation group (76.8%) [HR: 0.82, (0.63-1.06), p=0.136].

Immunotherapy

The GeparNuevo trial [17] evaluated early-stage TNBC patients without previous treatment to determine whether the addition of an anti-program death-ligand 1 (PD-L1) checkpoint inhibitor influenced complete response or survival. There was a modest improvement in pCR in the group treated with durvalumab (61%) vs. chemotherapy only (41%) [OR: 2.22 (1.06-4.64), p=0.035]; however, durvalumab combined with neoadjuvant chemotherapy significantly improved outcomes as DFS markedly improved in these patients [91.4% vs. 79.5%: HR: 0.37 (0.15-0.87), p=0.0148]. 

Overall, the results of these trials show that the addition of an immune checkpoint inhibitor increases the pCR, regardless of PD-L1 activity. The overall effect on survival may be significantly improved but requires continued follow-up. The landmark KEYNOTE trial [19] found that patients with early TNBC had a significantly higher pCR when treated with pembrolizumab plus neoadjuvant chemotherapy as opposed to neoadjuvant chemotherapy with placebo (64.8% vs. 51.2%, respectively; p<0.01). In early TNBC, this benefit was seen regardless of PD-L1 level, leading to the FDA approval of pembrolizumab for the treatment of mTNBC. The NeoTRIPaPDL1 [18] trial found that in patients with early-stage TNBC, when chemotherapy alone was evaluated against the addition of atezolizumab, there was no significant difference in the pCR (42.3% in placebo vs. 47.1% in atezolizumab group, p=0.66). PD-L1 expression was found to be significantly associated with the pCR but was highest in the “immune-rich” groups, or those tumors that overexpressed PD-L1 or had a higher proportion of tumor-infiltrating lymphocytes (TILs) [19]. In patients who had PD-L1 expression of at least 5%, the pCR was 55%, compared to 32% in those with PD-L1 expression between 1-5% (p=0.148).

Metastatic TNBC (mTNBC)

Chemoresistance and early recurrence remain predominant features of TNBC, especially in metastatic lesions. The current regimen in mTNBC is multimodal and dependent on both patient characteristics and mutational burden. Platinum-based therapies have shown mixed benefits in mTNBC patients (DFS: 7.8 months vs. 4.9 months, p<0.001; mOS: 19.6 months vs. 19.2 months, p=0.82) [16]. A comprehensive approach includes immunotherapy, ADC, and targeted therapies.

Role of Immunotherapy in mTNBC

The combined positive score (CPS) algorithm is a pathologic testing procedure developed for the evaluation of PD-L1 expression. CPS is determined by calculating the number of PD-L1 staining cells divided by the total number of viable tumor cells, multiplied by 100. The KEYNOTE [19] study assessed metastatic TNBC patients but allowed for a broad range of chemotherapeutic options in the stratification. Patients were randomized to either chemotherapy with pembrolizumab or chemotherapy with placebo. In the primary analysis of tumors regardless of mutation status, there was a mildly significant increase in PFS in the pembrolizumab group [7.5 months vs. 5.6 months; HR: 0.82 (0.69-0.97)]. When stratified, this improvement was similar in the PD-L1 negative group (7.6 months vs. 5.6 months; HR: 0.74, p<0.001), but markedly improved in the PD-L1 positive group (9.7 months vs. 5.6 months; HR: 0.60, p<0.01). However, due to the design of the trial, this finding was not considered statistically significant and pembrolizumab was only approved for patients with CPS >10.

TNBC remains a strong candidate for immunotherapy due to its high mutation complexity, availability of limited therapeutic options, and the presence of increased amounts of PD-1+ tumor-infiltrating lymphocytes (TILs). The IMpassion130 [20] trials studied metastatic or locally advanced TNBC patients who were treatment-naive in their metastatic setting and were randomized to either nab-paclitaxel with placebo vs. nab-paclitaxel with atezolizumab. The addition of atezolizumab in the PD-L1 positive tumors showed a significant increase in the PFS from 7.5 months vs. 5.3 months [HR: 0.63, (0.50-0.80), p<0.05] and marginally significant improvements in OS of 25 months vs. 18 months [HR: 0.71, (0.54-0.93), p<0.05]. These improvements were not observed in the PD-L1 negative population.

The IMpassion131 trial [21] had a similar design; however, the chemotherapy agent was paclitaxel in this group and was administered with concurrent dexamethasone. This study found no advantage with the addition of atezolizumab in PD-L1+ patients [22.1 vs. 28.3 months; HR: 1.12 (0.76-1.65)]. It is hypothesized that differences in the chemotherapy types, the heterogeneity of TNBC, the addition of steroids in the management, and unknown variables (i.e., antibiotic use/microbiome) may act as confounders for the association that was seen in the IMpassion130 trial. 

There are a variety of immune-specific adverse events that occur with the utilization of immune checkpoint inhibitors, which have been displayed in the treatment of TNBC. The most common adverse events tend to be dermatitis, endocrinopathies (hypo- or hyperthyroidism, diabetes), pneumonitis, autoimmune cytopenias, and hepatitis. However, severe-grade adverse events (grade 5) are relatively uncommon (less than 0.2%) [16-21].

Role of Antibody-Drug Conjugates in mTNBC

Trop-2 is a transmembrane glycoprotein that is upregulated in all cancer types and is associated with a poorer prognosis, which makes it an attractive chemotherapeutic target [25]. Sacituzumab govitecan (SG; Trodelvy) is an ADC highly specific for trop-2 and with a high drug:antibody ratio (7:1). This ADC contains a linker protein that triggers the release of a highly potent form of irinotecan named SN-38 upon intracellular hydrolysis [26].

SG received FDA approval in 2020 for use in the treatment of mTNBC previously treated with at least two prior chemotherapeutic agents. This recommendation was based on the ASCENT trial [27], which randomized patients into SG versus a treatment of physician’s choice (TPC). The patients were continued on therapy until progression or unacceptable toxicity. The trial was discontinued early due to significant improvement in the treatment arm. Notably, 30% of patients had previously received a checkpoint inhibitor. In patients treated with SG, the median PFS (mPFS) was 5.6 months vs. 1.7 months [HR: 0.41 (0.32-0.52), p<0.001]. The median OS (mOS) was significantly increased at 12.1 months vs. 6.7 months [HR: 0.48; (0.38-0.59), p<0.001] [27]. Adverse effects were common with nearly half of the population (46%) presenting with grade-3 neutropenia and 10% with diarrhea. Severe adverse effects (grade 4 or above) were less likely than in chemotherapy. Biomarker analysis [27,28] was performed to assess whether high expression of trop-2 was more likely to respond to SG therapy, but the objective response rate (ORR) showed that there was a significant benefit from SG despite trop-2 quantity. This effect was still present regardless of the germline BRCA status.

Role of PARP Inhibitors in mTNBC

PARP is an important regulator of DNA repair and has been shown to augment platinum-based chemotherapy [29-31]. O’Shaughnessy et al. [22] studied the effects of gemcitabine and carboplatin with or without augmentation with iniparib. More than half (52%) of the iniparib group achieved a pCR compared to 32% in the GC-only group (p=0.02). Additionally, iniparib increased DFS [5.9 months vs. 3.6 months; HR: 0.59 (0.39-0.90)] and OS [12.3 months vs. 7.7 months; HR: 0.57 (0.36-0.90)]. 

There are two major phase-III studies that have evaluated the use of PARP inhibitors in the treatment of advanced breast cancer [31,32]. The OlympiAD trial [24] randomized germline BRCA patients into olaparib or TPC, while the EMBRACA [23] trial randomized patients into talazoparib vs. TPC. In the OlympiAD trial, the eligible patients had received less than two prior chemotherapeutic regimens, while the EMBRACA trial allowed for less than three regimens. Both trials assessed the PFS of patients undergoing treatment.

In OlympiAD, there was no statistically significant improvement in the OS with olaparib compared to TPC. However, in a subset analysis, patients with no prior chemotherapy had a clear advantage in PFS with PARP inhibitors [HR: 0.51; (0.29-0.90), p=0.02]. However, those with prior chemotherapy had less of an impact [HR: 1.13; (0.79-1.64)], which may suggest that early use could generate a greater response. Similarly, the EMBRACA trial showed that patients who received PARP inhibitor therapy had significantly increased PFS of 8.6 months vs. 5.6 months [HR: 0.54; (0.41-0.71), p<0.001]. However, in the final OS analysis, there was no statistically significant difference between the two groups [19.3 months (talazoparib) vs. 19.5 months (TPC); HR: 0.848 (0.67-1.07), p=0.17]. When the investigators adjusted for subsequent PARP and/or platinum use, the HR for OS was 0.75 (0.503-1.029). While talazoparib did not significantly improve OS compared to TPC chemotherapy, subsequent treatments may have affected the analysis. Tung et al. [32] presented an abstract at the 2020 American Society for Clinical Oncology (ASCO), which suggested that patients with mTNBC that possess PALB2 mutations or somatic BRCA mutations have a significant response to olaparib (ORR: 82%, PALB2; ORR: 50%, sBRCA), which may allow for new treatment options in these subsets of patients.

Other targets

Role of Pan-AKT Inhibitors

The PI3K/AKT/mTOR signaling pathway is a complex intracellular biochemical cascade that is routinely disrupted in breast cancer and its activation favors cellular growth, proliferation, and survival [33,34]. Ipatasertib and Capivasertib are both pan-AKT inhibitors that are still under consideration for the treatment of mTNBC [35,36]. Both competitively inhibit all AKT isoforms and suppress the phosphorylation of AKT substrates that mediate cellular processes such as mitosis, apoptosis, and glucose or fatty acid metabolism.

LOTUS is a phase II clinical trial that compared Ipatasertib with paclitaxel against a placebo in mTNBC patients [35]. The intent-to-treat group that received the Ipatsertib/paclitaxel combination benefitted from a higher mPFS of 6.2 months compared to 4.9 months in the placebo group [HR: 0.60 (0.37-0.98), p=0.037]. Although these results were only marginally significant, a similar phenomenon was observed in 48 patients with PTEN-low tumors who shoed a higher mPFS while on Ipatsertib/paclitaxel compared to placebo [6.2 months vs. 3.7 months; HR: 0.59 (0.26-1.32), p=0.18]. The FAIRLANE trial also demonstrated that Ipatsertib had a pCR of 17%, which was higher than that in the 13% of patients in the placebo group [37].

In the PAKT trial, the mTNBC patients who received Capivasertib/paclitaxel had an mPFS of 5.9 months, while it was 4.2 months in the placebo group [HR: 0.74 (0.50-1.08), one-sided p=0.06] [36]. Furthermore, the Capivasertib/paclitaxel group also had a higher mOS than the placebo group [19.1 months vs. 12.6 months; HR: 0.30 (0.11-0.79), two-sided p=0.01].

Role of Bromodomain Inhibitors in mTNBC

Bromodomain-containing proteins (BCPs) are enzymes that modulate the transcription of various genes by binding to the acetylated lysine residues of histones [38,39]. The BRD2, BRD3, BRD4, and BRDT proteins are a part of the bromodomain and extraterminal (BET) domain family, which are responsible for the regulation of various physiologic functions from cardiovascular health to inflammation and have shown great promise in the treatment of mTNBC [40,41]. 

GSK-2801 is a BAZ3/BRD9 bromodomain inhibitor that is currently being studied in cellular cultures [41]. Bevill et al. have observed that GSK-2801 thoroughly silenced the transcription of ribosomal DNA and the expression of ETS-regulated genes, which forced mTNBC cells to undergo apoptosis [41]. The bromodomain inhibitor OTX015 (Birabresib) has also shown antiproliferative properties in TNBC-derived cell lines [42]. This inhibitor has been shown to induce cell cycle arrest after 72 hours, decrease c-Myc expression, and affect cancer stem cells. A phase-I clinical trial evaluating the effects of Birabresib is currently underway [43]. 

Role of Aurora Kinase Inhibitors in mTNBC

In human cells, the Aurora kinase family represents highly conserved serine/threonine protein kinases; it was first discovered in the early 1990s and has been shown to tightly control several mitotic events [44,45]. Recent studies have uncovered that Aurora kinase dysregulation is associated with carcinogenesis and confers tumor cell radio- and chemoresistance [46]. ENMD-2076 and alisertib are Aurora kinase inhibitors that are currently under investigation for the treatment of TNBC [47,48].

A phase-II clinical trial evaluating the effects of ENMD-2076 in 41 TNBC patients revealed that the clinical benefit rate (CBR) at four months was 27.8%, and it was 16.7% at six months [47]. The average duration of benefit was determined to be 6.5 cycles. In mTNBC, alisertib has shown increased mPFS compared to the paclitaxel group [10.2 months vs. 7.1 months; HR: 0.56 (0.37-0.84), p=0.005]. Alisertib was also associated with a higher mOS than paclitaxel alone [26.3 months vs. 25.1 months; HR: 0.89 (0.58-1.38), p=0.61], albeit it was not statistically significant [48]. 

Role of CHK1 Inhibitors in mTNBC

Checkpoint kinase-1 (CHK-1) is a serine/threonine kinase that plays an important role in the DNA damage response (DDR) pathway by facilitating cell cycle arrest [49]. Liu et al. revealed that CHK-1 acts as a cell cycle checkpoint control with the help of ATR once exposed to ultraviolet radiation, gamma-radiation, or hydroxyurea [50]. In comparison, the ATM-CHK-2 pathway is activated in response to DNA double-stranded breaks [50]. Surprisingly, CHK-1 expression patterns are increased in TNBC and are thought to be associated with tumor grade and disease recurrence [49,51,52]. Prexasertib, UCN-01, GDC-0425, and MK-8776 are some of the CHK-1 inhibitors that are currently under evaluation for TNBC [53-56].

A phase-II single-arm pilot study of prexasertib showed that its ORR was 11.1% in nine patients diagnosed with TNBC [53]. Four of these patients had stable disease at follow-up with an mPFS of 86 days. Prexasertib-treated cells experienced a 55% reduction in homologous recombination [53]. UCN-01 has also shown promising results in a phase-II study with a CBR of 12% and ORR of 4% [54].

Future directions

Cancer Vaccines With or Without PD-L1 Inhibitors

The discovery of the increased immunogenicity of TNBC tumors and their association with TILs has led to the advent of cancer vaccination to generate tumor-specific immunity and prevent disease recurrence [57]. A summary of the current clinical trials evaluating these therapies in TNBC is presented in Table 3.

Some types of TNBC, termed HER2-low, express low levels of HER2, albeit not enough for targeted therapy [58]. AE37 is an MHC class-II peptide that is derived from HER2 and is thought to promote the increase of TILs that may target these tumors. A phase-II trial evaluating the use of the AE37 peptide vaccine showed improvement in DFS among mTNBC patients [59]. This led to the NSABP FB-14 Phase-II (NCT04024800) [60] trial, which is currently studying AE37 peptide vaccines with pembrolizumab to enhance tumor-specific immune responses in mTNBC. In other studies, the combination of a HER2-derived peptide vaccine, nelipepimut-S (NPS), with trastuzumab induced a strong T-cell response, which correlated to improvements in DFS when compared to trastuzumab alone [61]. Finally, PVX-410 is an HLA A2-restricted cancer vaccine that has shown significant improvements in smoldering multiple myeloma [62]. The phase-I trial (NCT03362060) is currently evaluating PVX-410 with pembrolizumab in metastatic patients [63].

Tumor-specific neoantigens are derived from tumor somatic mutations and chromosomal rearrangements [64,65]. These neoantigens have been studied in RNA immunotherapy and their use in mRNA vaccines is a new and bright focus in the development of personalized cancer treatment [66]. The phase-I TNBC-MERIT trial (NCT02316457) [67] is currently studying RNA immunotherapy by utilizing two methods: the WAREHOUSE approach and the IVAC MUTANOME concept. The WAREHOUSE approach treats patients with a selected group of breast cancer-associated antigens that have proven immunogenicity. The MUTANOME arm targets multiple neoantigens from mutated epitopes with the idea that mutation-specific T cells bear enormous potential for anti-tumor activity. Turner et al. [68] and Gillanders et al. [69] are also studying circulating tumor DNA (ctDNA) or neoantigens in vaccines to determine how their effect on standard therapies with or without monoclonal antibodies affect patient outcomes. 

Anti-Androgen Hormonal Therapy

TNBCs may express other hormone receptors such as androgen receptors (AR). In fact, the AR is expressed in approximately 30% of all TNBC and its inhibition has been shown to reduce proliferation, migration, and invasion of TNBC cell lines in vitro [70]. Enzalutamide is a promising new targeted therapy that is currently being studied in a phase II trial (NZCT01889238) for the treatment of AR+ TNBC [71]. Additionally, the nonsteroidal AR inhibitor bicalutamide, which is currently used in locally advanced and metastatic prostate cancer, is currently being studied in a phase II trial (NCT03090165) for the treatment of TNBC [72].

Table 3 provides a summary of the select ongoing clinical trials in TNBC.

**Table 3:** Selection of current clinical trials in triple-negative breast cancer mTNBC: metastatic triple-negative breast cancer; ORR: overall response rate; ctDNA: circulating tumor DNA; AR: androgen receptor

Clinical trial ID #	Phase	Patients	Interventions	Outcomes of interest
NCT04024800 [60]	II	mTNBC (n=29)	AE37 peptide vaccine +/- GMCSF + pembrolizumab	Recommended dose, ORR
NCT03362060 [63]	I	HLA-A2+ mTNBC (n=20)	PVX-410 vaccine + pembrolizumab	Immune response, ORR
NCT02316457 [67]	I	TNBC (n=42)	IVAC_W_bre1_uID vaccination	Number of adverse events, induced T-cell response
NCT03145961 [68]	II	Early-stage TNBC (n=208)	Pembrolizumab	ctDNA levels
NCT03199040 [69]	I	TNBC (n=13)	Neoantigen DNA vaccine +/- durvalumab	Safety immune response
NCT01889238 [71]	II	Advanced AR+ TNBC (n=118)	Enzalutamide	Clinical benefit rate
NCT03090165 [72]	I/II	AR+ TNBC (n=37)	Ribociclib + bicalutamide	Maximum tolerated dose, clinical benefit rate, ORR

Limitations

This study has a few limitations. The articles utilized for this review were extracted from only two separate databases. which may have led to the possibility of publication bias. However, the goal of this narrative review was to organize and summarize the latest studies that have impacted the clinical management of both early- and late-stage TNBC. The results of comparisons between trials must be analyzed with the understanding that the patient populations studied were diverse as they differed in terms of functional status and previous therapies. This makes the extrapolation of these findings to the general population difficult. However, we feel that we have identified specific subgroups of TNBC that could benefit from individual therapies and have highlighted the studies that show these benefits.

## Conclusions

Patients with early triple-negative breast cancer retain the benefit from standard anthracycline/taxane regimens. The use of platinum-based therapies may enhance pCR but it has not been shown to improve survival. Patients with residual disease after neoadjuvant chemotherapy and surgical management may benefit from adjuvant capecitabine. The addition of immunotherapy with checkpoint inhibitors may enhance pCR in the neoadjuvant setting, especially in those who overexpress PD-L1 or have high numbers of TILs. mTNBC patients have shown improved survival when treated with immunotherapy as their high mutation burden correlates with increased immunogenicity. ADCs such as sacituzumab govitecan may provide additional benefits for patients who have progressed through other therapies. PARP inhibitors can improve survival in patients with germline BRCA mutations but show the most benefit in those without prior treatment. However, PARP inhibitors may have a selective advantage in those with somatic BRCA or PALB2 mutations. Novel targets such as pan-AKT inhibitors, bromodomain inhibitors, aurora kinase inhibitors, and CHK1-inhibitors have shown promising results as adjuvants to chemotherapy, but these trials are still in their early stages.

The future of TNBC treatment may lie in two well-studied options: vaccination and hormonal blockade. The immunogenic tumor microenvironment responds well to immunotherapy; however, the results have been more significant in PD-L1-positive tumors. The vaccination of patients with tumor neoantigens may increase the immunogenicity of these patients, thereby making them responsive to proven immunotherapies. While being historically low-positive or negative for estrogen and progesterone receptors, nearly one-third of TNBC patients express ARs and may benefit from targeted androgen blockade with well-studied prostate cancer treatments.
